# The impacts of cytoplasmic incompatibility factor (*cifA* and *cifB*) genetic variation on phenotypes

**DOI:** 10.1093/genetics/iyaa007

**Published:** 2020-11-20

**Authors:** J Dylan Shropshire, Rachel Rosenberg, Seth R Bordenstein

**Affiliations:** 1 Department of Biological Sciences, Vanderbilt University, VU Station B, Box 35-1634, Nashville, TN 37235, USA; 2 Vanderbilt Microbiome Initiative, Vanderbilt University, VU Station B, Box 35-1634, Nashville, TN 37235, USA; 3 Division of Biological Sciences, University of Montana, 32 Campus Drive, Missoula, MT 59812, USA; 4 Department of Pathology, Microbiology, and Immunology, Vanderbilt University, Nashville, TN 37235, USA; 5 Vanderbilt Institute for Infection, Immunology, and Inflammation, Vanderbilt University Medical Center, Nashville, TN 37235, USA

**Keywords:** cytoplasmic incompatibility, *Wolbachia*, phage WO, *Drosophila melanogaster*, Two-by-One model

## Abstract

*Wolbachia* are maternally transmitted, intracellular bacteria that can often selfishly spread through arthropod populations via cytoplasmic incompatibility (CI). CI manifests as embryonic death when males expressing prophage WO genes *cifA* and *cifB* mate with uninfected females or females harboring an incompatible *Wolbachia* strain. Females with a compatible *cifA*-expressing strain rescue CI. Thus, *cif-*mediated CI confers a relative fitness advantage to females transmitting *Wolbachia*. However, whether *cif* sequence variation underpins incompatibilities between *Wolbachia* strains and variation in CI penetrance remains unknown. Here, we engineer *Drosophila melanogaster* to transgenically express cognate and non-cognate *cif* homologs and assess their CI and rescue capability. Cognate expression revealed that *cifA;B* native to *D. melanogaster* causes strong CI, and cognate *cifA;B* homologs from two other *Drosophila*-associated *Wolbachia* cause weak transgenic CI, including the first demonstration of phylogenetic type 2 *cifA;B* CI. Intriguingly, non-cognate expression of *cifA* and *cifB* alleles from different strains revealed that *cifA* homologs generally contribute to strong transgenic CI and interchangeable rescue despite their evolutionary divergence, and *cifB* genetic divergence contributes to weak or no transgenic CI. Finally, we find that a type 1 *cifA* can rescue CI caused by a genetically divergent type 2 *cifA;B* in a manner consistent with unidirectional incompatibility. By genetically dissecting individual CI functions for type 1 and 2 *cifA* and *cifB*, this work illuminates new relationships between *cif* genotype and CI phenotype. We discuss the relevance of these findings to CI’s genetic basis, phenotypic variation patterns, and mechanism.

## Introduction

*Wolbachia* are intracellular bacteria that occur in 40–65% of arthropod species (Hilgenboecker *et al.* 2008; Zug and Hammerstein 2012; Weinert *et al.* 2015; Charlesworth *et al.* 2019). While often horizontally transmitted between species (Boyle *et al.* 1993; Huigens *et al.* 2004; Gerth *et al.* 2013; Tolley *et al.* 2019; Scholz *et al.* 2020), vertical transmission from mother to offspring predominates within species (Turelli and Hoffmann 1991; Narita *et al.* 2009). *Wolbachia* can frequently increase their rate of spread in host populations through the matriline by causing cytoplasmic incompatibility (CI). CI results in embryonic death of uninfected embryos after fertilization by *Wolbachia*-modified sperm (Yen and Barr 1973; Shropshire *et al.* 2020b). Embryos with compatible *Wolbachia* are rescued from CI-induced lethality, yielding a relative fitness advantage to *Wolbachia*-infected females that transmit the bacteria to their offspring (Hoffmann *et al.* 1990; Turelli 1994; Turelli and Hoffmann 1995). CI frequently manifests between arthropods infected with different *Wolbachia* strains. Strains may be reciprocally incompatible (bidirectional CI), or only one of the two strains can rescue the other’s sperm modification (unidirectional CI). CI-inducing *Wolbachia* have garnered attention for their role in suppressing vector populations (Lees *et al.* 2015; Nikolouli *et al.* 2018; Crawford *et al.* 2020), curbing the transmission of pathogenic RNA viruses (O’Neill 2018; Moretti *et al.* 2018; Gong *et al.* 2020), and reproductive isolation and incipient speciation (Bordenstein *et al.* 2001; Jaenike *et al.* 2006; Brucker and Bordenstein 2012; Shropshire and Bordenstein 2016).

**Figure 1:**
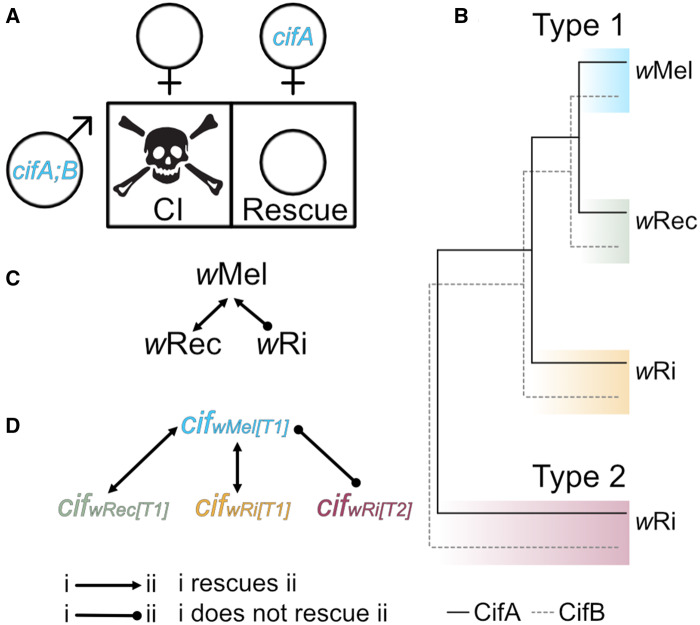
Two-by-One model, Cif phylogeny, and predicted relationships between *w*Mel, *w*Rec, and *w*Ri strains and *cif* gene variants. (A) The Two-by-One genetic basis of CI: *cifA;B* causes CI that can be rescued by females expressing *cifA* (Shropshire and Bordenstein 2019). (B) Schematic representation of the evolutionary relationships between CifA and CifB proteins from *w*Mel, *w*Rec, and *w*Ri (LePage *et al.* 2017). (C) Incompatibilities between *w*Mel, *w*Rec, and *w*Ri *Wolbachia* strains. Unidirectional CI between *w*Mel and *w*Ri is based on crossing experiments after the transinfection of *w*Mel into *D. simulans* (Poinsot *et al.* 1998). Compatibility between *w*Mel and *w*Rec is based on the prediction that strains with closely related *cif* gene sequences are compatible. (D) Predicted incompatibility relationships between *cif* homologs from each of the three strains, based on sequence relationship. Lines between strains/genes indicate compatibility relationships. If the line ends in an arrowhead, then the strain/gene(s) at the beginning of the arrow can rescue CI caused by the strain/gene(s) the arrow points toward. If the line ends in a circle, then rescue is not expected. Skull art is modified from vecteezy.com with permissions.

Two adjacent genes in the eukaryotic association module of *Wolbachia*’s prophage WO cause CI when expressed in males (*cifA* and *cifB*) (Bordenstein and Bordenstein 2016; Beckmann *et al.* 2017; LePage *et al.* 2017; Chen *et al.* 2019; Shropshire and Bordenstein 2019), and one of the same genes rescues CI when expressed in females (*cifA*) (Shropshire *et al.* 2018; Chen *et al.* 2019; Shropshire and Bordenstein 2019). These results established the Two-by-One genetic model of CI (Figure 1A) (Shropshire and Bordenstein 2019), but its generality across *cif* homologs remains to be evaluated. Singly expressing a small set of *cifA* variants that have only annotated domains or *cifB* variants that exhibit *in vitro* deubiquitilase and nuclease activities also does not cause rescuable embryonic death (Beckmann *et al.* 2017; LePage *et al.* 2017; Chen *et al.* 2019; Shropshire and Bordenstein 2019). Cif proteins segregate into at least five phylogenetic clades (types 1–5) (LePage *et al.* 2017; Lindsey *et al.* 2018; Bing *et al.* 2020; Martinez *et al.* 2020), and distant Cif-like homologs are found in *Orientia* and *Rickettsia* bacteria, which are not known to cause CI (Gillespie *et al.* 2018). To date, the genetic basis for CI (Figure 1A) has been tested using *cif* transgenes from the types 1 and 4 clades in *w*Mel *Wolbachia* of *Drosophila melanogaster* and *w*Pip *Wolbachia* of *Culex pipiens* mosquitoes (Beckmann *et al.* 2017; Chen *et al.* 2019; Shropshire and Bordenstein 2019). Thus, a considerable amount of phylogenetic diversity remains untested.

Moreover, while the genetic basis of CI between infected and uninfected insects is resolved for some strains, the genetic basis of unidirectional or bidirectional CI between insects harboring different *Wolbachia* strains remains largely unknown. Phylogenetic and sequence analyses of *cif* genes from incompatible *Wolbachia* strains in *Drosophila* or *Culex* reveal that incompatible *Wolbachia* strains differ in genetic relationship and/or copy number (Bonneau *et al.* 2018, 2019; LePage *et al.* 2017), supporting *cif* variation as the basis of strain incompatibilities. Moreover, since *cifA* is involved in both CI induction and rescue, a single-step evolutionary model for bidirectional CI has been proposed where a single mutation in *cifA* leads to incompatibility between the ancestral and derived variants while retaining compatibility with the emergent variant and requiring *cifB* only as an accessory function (Shropshire *et al.* 2018). However, these hypotheses have not been empirically tested.

In this study, we first test *cif* homologs from *w*Mel of *D. melanogaster*, *w*Rec of *D. recens*, and *w*Ri of *D. simulans* for CI and rescue when transgenically expressed in uninfected *D. melanogaster*. We previously determined that *w*Mel genes adhere to the Two-by-One model (Shropshire and Bordenstein 2019), but the genetic bases of *w*Rec and *w*Ri CI remain unknown. *w*Rec and *w*Ri are strong CI inducers that cause high degrees of embryonic death (Turelli and Hoffmann 1991; Werren and Jaenike 1995; Shoemaker *et al.* 1999). Both encode phylogenetic type 1 *cif* genes similar to *w*Mel, and *w*Ri also encodes a type 2 *cif* pair that is highly diverged from *w*Mel (Figure 1B) (LePage *et al.* 2017). Like *w*Mel, we predict that *w*Rec and *w*Ri CI have a Two-by-One genetic basis. This is the first time type 2 *cif* genes have been functionally interrogated.

Next, we test the crossing relationships between divergent *cif* homologs to investigate the basis of interstrain incompatibilities. *w*Ri and *w*Mel *Wolbachia* are unidirectionally incompatible in a common *D. simulans* host background. In other words, *w*Ri can rescue *w*Mel-induced CI, but *w*Mel cannot rescue *w*Ri-induced CI (Figure 1C) (Poinsot *et al.* 1998). Thus, we hypothesize that *w*Ri can rescue *w*Mel-induced CI because it has type 1 *cif* genes comparable to *w*Mel, and *w*Mel cannot rescue *w*Ri because it does not have genes capable of rescuing *w*Ri’s type 2 genes (Figure 1D) (LePage *et al.* 2017). Moreover, since *w*Rec’s type 1 *cif* genes are closely related to *w*Mel genes, we predict them to be compatible upon transgenic expression (Figure 1C and D). We discuss our results in the context of the genetic basis of CI in these strains, the causes of CI strength variation and strain incompatibilities, and CI’s molecular basis.

## Materials and Methods

### Fly lines and maintenance

The following Upstream Activation Sequence (UAS) transgenic constructs were generated for this study: *cifA_wRec[T1]_*, *cifB_wRec[T1]_*_,_*cifA_wRi[T1]_*_,_*cifB_wRi[T1;N]_*, *cifB_wRi[T1;C]_*, *cifB_wRi[T1;poly]_*, *cifA_wRi[T2]_*, and *cifB_wRi[T2]_*. Each transgene was codon-optimized for expression in *D. melanogaster* and synthesized by GenScript (Hong Kong, China). Valine start codons were replaced with methionine. Wild-type and codon-optimized gene sequences are reported in Supplementary Table S2. Each gene was cloned into the pTIGER plasmid at GenScript. pTIGER is a pUASp-based vector designed for germline expression and was previously used to generate *cifA_wMel[T1]_* and *cifB_wMel[T1]_* transgenes also used in this study (LePage *et al.* 2017). pTIGER enables phiC31 integration into the *D. melanogaster* genome, contains a UAS promoter region intended for GAL4/UAS expression, and has a red-eye marker for screening. *D. melanogaster* embryo injections were conducted by Best Gene (Chino Hills, California) using phiC31 integrase to place *cifA* and *cifB* homologs into the Attp40 (chromosome 2) and Attp2 (chromosome 3) insert sites, respectively. Transformants were screened via eye color, and homozygous transgenic lines were generated for all lines. All lines were negative for *Wolbachia* based on PCR using Wolb_F and Wolb_R3 primers (Casiraghi *et al.* 2005). Dual expressing UAS transgenic lines were generated via standard genetic crossing schemes.

In addition, the following *D. melanogaster* stocks were used in this study: infected and uninfected *y*^1^w* (BDSC 1495), uninfected *nos*-GAL4:VP16 (BDSC 4937), and uninfected UAS transgenic lines homozygous for *cifA_wMel[T1]_*, *cifB_wMel[T1]_*, and *cifA;B_wMel[T1]_* (LePage *et al.* 2017). Genotypes and infection states were regularly confirmed for transgene expressing fly lines using primers listed in Supplementary Table S3. *D. melanogaster* stocks were maintained at 12:12 light:dark at 25°C on 50 ml of a standard media. Stocks for virgin collections were stored at 18°C overnight to slow eclosion rate, and virgin flies were kept at room temperature.

### Hatch rate assays

To test for CI, hatch rate assays were conducted as previously described (LePage *et al.* 2017; Shropshire *et al.* 2018). Briefly, virgin *nos*-GAL4:VP16 adult females were aged 9–11 days post-eclosion, to control for the paternal grandmother age effect (Layton *et al.* 2019), and mated with UAS transgenic or *y*^1^w* males. GAL4-UAS males and females were paired in 8-oz round bottom *Drosophila* bottles (Genesee Scientific) affixed with a grape-juice agar plate smeared with yeast affixed to the opening with tape. To control the impact of male age and the younger brother effect on CI level (Reynolds and Hoffmann 2002; Yamada *et al.* 2007), only young early emerging males (0–48 h) were used in crossings. Conversely, 5–7-day-old females were used since they are highly fecund. The flies and bottles were stored at 25°C for 24 h at which time the plates were replaced with freshly smeared plates and again stored for 24 h. Plates were then removed, and the number of embryos on each plate was counted and stored at 25°C. After 30 h, the remaining unhatched embryos were counted. The percentage of embryos that hatched into larvae was calculated by dividing the number of hatched embryos by the initial embryo count and multiplying by 100.

### Embryonic cytology

Flies were collected, aged, and crossed as described for hatch rate assays. However, 60 females and 12 males were included in each bottle with a grape-juice agar plate attached. Flies were siblings of those in hatch rate assays. Embryos laid in the first 24 h were discarded due to low egg-laying. During the second day, embryos were aged 1–2 h and then dechorionated, washed, and fixed in methanol as previously described (LePage *et al.* 2017; Shropshire *et al.* 2018). Embryos were stained with propidium iodide and imaged (LePage *et al.* 2017; Shropshire *et al.* 2018). Scoring of cytological defects was conducted using previously defined characteristics (LePage *et al.* 2017).

### Sequence analyses

Sequence similarity between Cif proteins was determined using pairwise MUSCLE alignments of protein sequences using default settings. Glimmer 3 was used to identify open reading frames in *cifB_wRi[T1]_* after the early stop codon that truncates the gene. These analyses were conducted in Geneious Prime.

### Statistical analyses

All statistical analyses were conducted in GraphPad Prism 8. Hatch rate statistical comparisons were made using Kruskal–Wallis followed by a Dunn’s multiple comparison test. Samples with fewer than 25 embryos laid were removed from hatch rate analyses as previously described (LePage *et al.* 2017). Hatch rates in main text figures display the combination of two replicate experiments, which were analyzed simultaneously, and those in the supplement display only single experiments (*N* = 8–58 per cross after exclusion). Replicate data were statistically comparable in all cases. Cytological abnormalities were compared using a pairwise Fisher’s exact test followed by a Bonferroni–Dunn correction test (*N* = 43–167 embryos per cross). Figure esthetics were edited in Affinity Designer 1.7 (Serif Europe, Nottingham, UK). All *P*-values are reported in Supplementary Table S1, and the exact sample sizing information is available in Supplementary File S1.

### Data availability

All data are made publicly available in the supplement of this manuscript. Fly lines not otherwise available in the Bloomington *Drosophila* Stock Center are available upon request.

Supplemental material is available at figshare DOI: https://doi.org/10.25386/genetics.13215503.

## Results

To distinguish between different *cifA* and *cifB* genetic variants, we use a gene nomenclature that identifies the *Wolbachia* strain in subscript and the *cif* phylogenetic type associated with the variant in brackets (Shropshire *et al.* 2019), following published standards (The Journal of Bacteriology 2018). For instance, *cif* genes of the *w*Mel strain belong to the type 1 clade and are referred to as *cifA_wMel[T1]_* and *cifB_wMel[T1]_*. We used the GAL4-UAS system (Duffy 2002) to drive the germline expression of *cif* transgenes in *D. melanogaster*, and all transgenes are expressed in uninfected flies using the *nos*-GAL4:VP16 driver that causes strong *cif_wMel[T1]_* CI and rescue (Shropshire and Bordenstein 2019). We measure CI as the percentage of embryos that hatch into larvae relative to a compatible control in which *cifA;B_wMel[T1]_* CI from males is rescued by *cifA_wMel[T1]_* females (LePage *et al.* 2017; Shropshire *et al.* 2018; Shropshire and Bordenstein 2019). This cross is included in all experiments and will hereafter be referred to as the “compatible control”. All protein annotations are derived from prior works (Lindsey *et al.* 2018).

### *Do phylogenetic type 1* cif *genes from* w*Rec transgenically induce and rescue CI?*

**Figure 2:**
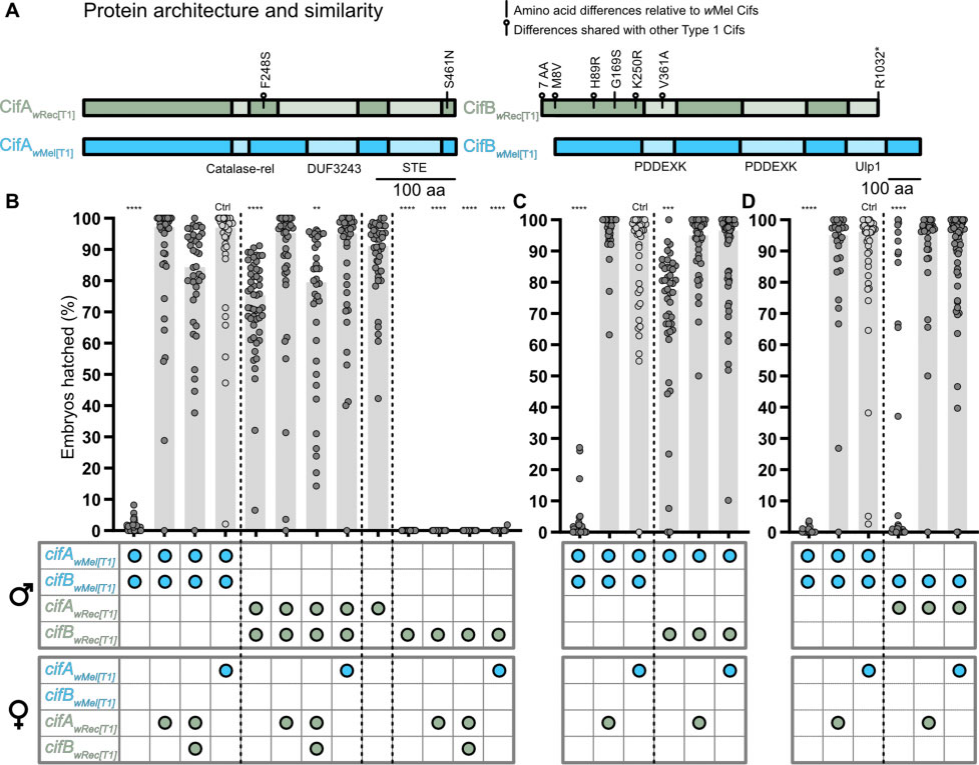
Cif_*w*Rec[T1]_ protein similarity and results of transgenic crosses including Cif_*w*Rec[T1]_ proteins. (A) Protein architecture of Cif_*w*__Mel[T1]_ and Cif_*w*__Rec[T1]_ (Lindsey *et al.* 2018). Substitutions inside schematics represent sequence identity relative to Cif_*w*__Mel[T1]_. Substitutions marked with a circle above the protein schema are shared between Cif_*w*__Rec[T1]_ and Cif_*w*__Ri[T1]_. R1032* represents an arginine to stop codon mutation. Hatch rate analyses testing (B) *cifA_wRec[T1]_*, *cifB_wRec[T1]_*, and *cifA;B_wRec[T1]_* for CI and rescue (*N* = 12–51 where each dot represents a clutch of embryos from a single mating pair), (C) *cifA_wMel[T1]_;cifB_wRec[T1]_* for CI (*N* = 36–55), and (D) *cifA_wRec[T1]_;cifB_wMel[T1]_* for CI (*N* = 27–58). Horizontal bars represent median embryonic hatching from single pair matings. Genotypes for each cross are illustrated below the bars where the genes expressed in each sex are represented by colored circles. Blue circles represent *cif_wMel[T1]_* genes, and green circles represent *cif_wRec[T1]_* genes. Each hatch rate contains the combined data of two replicate experiments, each containing all crosses shown. Asterisks above bars represent significant differences relative to a control transgenic rescue cross (denoted Ctrl) with an α = 0.05. *P < 0.05, **P < 0.01, ***P < 0.001, ****P < 0.0001. Exact *P*-values are provided in Supplementary Table S1.

Relative to Cif_*w*Mel[T1]_ proteins, CifA_*w*__Rec[T1]_ has two amino acid substitutions in unannotated regions: one prior to CifA’s putative DUF3243 and another after the annotated STE domain (Figure 2A). CifB_*w*__Rec[T1]_ has 13 amino acid changes that include a seven amino acid extension on the N-terminus, four substitutions in the N-terminal unannotated region, a single substitution in the first putative PD-(D/E)XK-like nuclease domain (hereafter PDDEXK), and a stop codon that truncates amino acids at residues 1032–1173 on the C-terminus of the protein (Figure 2A). *w*Rec causes strong CI in *D. recens* (Shoemaker *et al.* 1999; Werren and Jaenike 1995), the *w*Rec genome lacks other *cif* genes (Metcalf *et al.* 2014), and these variants are highly similar to *cif_wMel[T1]_* genes (Figure 2A). Thus, we predicted that *cifA;B_wRec[T1]_* expression in uninfected males will cause CI, transgenic *cifA_wRec[T1]_* expression in uninfected females will rescue CI, and CI induced by *cif_wRec[T1]_* transgenes will be compatible with *cif_wMel[T1]_* transgenes.

Consistent with prior reports in *D. melanogaster*, *cifA;B_wMel[T1]_* males induce strong CI that is rescued by the compatible control cross with *cifA_wMel[T1]_* females (Figure 2B) (Shropshire and Bordenstein 2019). *cifA;B_wRec[T1]_* males also cause a small but statistically significant reduction in hatching (Mdn = 75.4% hatching; *P* < 0.0001; Figure 2B) that is rescued by *cifA_wRec[T1]_* females but not by *cifA;B_wRec[T1]_* females (Mdn = 79.6% hatching; *P* = 0.0054). Results therefore suggest that *cifA_wRec[T1]_* is a rescue gene, weak *cifA;B_wRec[T1]_* CI is rescuable, and *cifB_wRec[T1]_* may reduce *cifA_wRec[T1]_* rescue capacity in embryos. Since neither *cifA_wMel[T1]_* nor *cifB_wMel[T1]_* induces CI alone (LePage *et al.* 2017; Shropshire and Bordenstein 2019), we predicted neither *cifA_wRec[T1]_* nor *cifB_wRec[T1]_* would reduce hatching. Indeed, *cifA_wRec[T1]_* males did not reduce hatching (*P* > 0.99). However, *cifB_wRec[T1]_* males caused complete embryonic death (Mdn = 0% hatching; *P* < 0.0001; Figure 2B) that was not rescued by *cifA_wRec[T1]_* (Mdn = 0% hatching), *cifA;B_wRec[T1]_* (Mdn = 0% hatching), *cifA_wMel[T1]_* (Mdn = 0% hatching), or *w*Mel-infected (Mdn = 0% hatching) females (Figure 2B and Supplementary Figure S1). Embryos fertilized by *cifB_wRec[T1]_* males had an unusually high percentage of early mitotic failures and single puncta indicative of unfertilized embryos or embryos undergoing mitotic failure in the first division (Supplementary Figure S2). However, unlike *cifA;B_wMel[T1]_* males, there were no later stage regional mitotic failures or chromatin bridging phenotypes, and the *cifB_wRec[T1]_* defects were unrescuable (Supplementary Figure S2) (LePage *et al.* 2017). Taken together, these results indicate that *cifB_wRec[T1]_* alone causes an atypical embryonic lethality relative to *cifA;B_wMel[T1]_*-induced CI.

Next, we tested crossing relationships between *cif_wMel[T1]_* and *cif_wRec[T1]_* transgenic males and females. Weak *cifA;B_wRec[T1]_*-induced CI was reduced when mated with *cifA_wMel[T1]_* females (*P* > 0.99) relative to the compatible control. Similarly, *cifA;B_wMel[T1]_* CI was reduced when mated with *cifA_wRec[T1]_* (*P* > 0.99) or *cifA;B_wRec[T1]_* (*P* = 0.10) females (Figure 2B). However, *cifA;B_wRec[T1]_* females only partially rescue *cifA;B_wMel[T1]_* CI, and since *cifA;B_wRec[T1]_* females do not rescue *cifA;B_wRec[T1]_* CI (Figure 2B), a firm conclusion cannot be made on whether *cifA;B_wRec[T1]_* females can rescue *cifA;B_wMel[T1]_* CI. Together, these data indicate that *cifA_wMel[T1]_* and *cifA_wRec[T1]_*, which differ by two amino acid substitutions in the putative DUF3243 and STE domains (Figure 2A), rescue the other strain’s transgenic CI. This is perhaps unsurprising since prior mutagenesis assays suggest conserved sites in DUF3243 and STE domains are not related to rescue (Shropshire *et al.* 2020a).

Finally, since *cifA;B_wRec[T1]_* males induce weak CI relative to *cifA;B_wMel[T1]_* males, we hypothesized that *cifA_wRec[T1]_* or *cifB_wRec[T1]_* sequence variation underpins CI level variation. We tested this hypothesis by engineering and expressing non-cognate combinations of *cif_wRec[T1]_* and *cif_wMel[T1]_* transgenes. We report that *cifA_wMel[T1]_*;*cifB_wRec[T1]_* males cause a weak but statistically significant reduction in hatching relative to the compatible control (Mdn = 77.6% hatching; *P* = 0.0008; Figure 2C), and this hatch rate reduction was comparable to that of cognate *cifA;B_wRec[T1]_* (Mdn = 75.4% hatching; Figure 2B) and likewise rescued when crossed to *cifA_wMel[T1]_* (*P* > 0.99) or *cifA_wRec[T1]_* (*P* > 0.99) females (Figure 2C). In contrast, *cifA_wRec[T1]_*;*cifB_wMel[T1]_* males caused strong CI (Mdn = 0% hatching; *P* < 0.0001) that was also rescued by *cifA_wRec[T1]_* (Mdn = 97.1% hatching; *P* > 0.99) or *cifA_wMel[T1]_* (Mdn = 95.9% hatching; *P* > 0.99) females (Figure 2D). These data demonstrate that the two closely related *cifA* sequences are interchangeable and fully capable of CI and rescue and that sequence variation in *cifB* is crucially responsible for weak *cifA;B_wRec_*_[T1]_ transgenic CI in *D. melanogaster*.

### *Do phylogenetic type 1* cif *genes from* w*Ri transgenically induce and rescue CI?*

**Figure 3:**
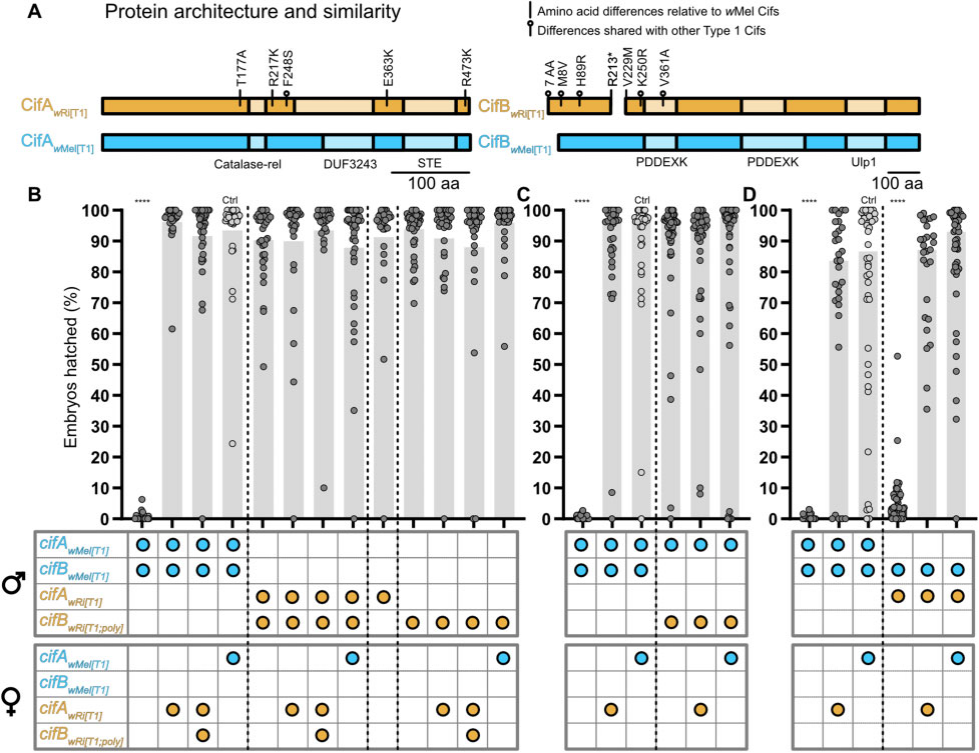
Cif_*w*Ri[T1]_ protein similarity and results of transgenic crosses including Cif_wRi[T1]_ proteins. (A) Protein architecture of Cif_*w*__Mel[T1]_ and Cif_*w*__Ri[T1]_ (Lindsey *et al.* 2018). Substitutions inside schematics represent sequence identity relative to Cif_*w*__Mel[T1]_. Substitutions marked with a circle above the protein schema are shared between Cif_*w*__Rec[T1]_ and Cif_*w*__Ri[T1]_. R213* represents an arginine to stop codon mutation. Hatch rate analyses testing (B) *cifA_wRi[T1]_*, *cifB_wRi[T1;poly]_*, and *cifA;B_wRi[T1;poly]_* for CI and rescue (*N* = 26–44 where each dot represents a clutch of embryos from a single mating pair), (C) *cifA_wMel[T1]_;cifB_wRi[T1;poly]_* for CI (*N* = 32–56), and (D) *cifA_wRi[T1]_;cifB_wMel[T1]_* for CI (*N* = 27–47). Horizontal bars represent median embryonic hatching from single pair matings. Genotypes for each cross are illustrated below the bars where the genes expressed in each sex are represented by colored circles. Blue circles represent *cif_wMel[T1]_* genes and orange circles represent *cif_wRi[T1]_* genes. All flies were uninfected with *Wolbachia*. Each hatch rate contains the combined data of two replicate experiments, each containing all crosses shown. Asterisks above bars represent significant differences relative to a control transgenic rescue cross (denoted Ctrl) with an α = 0.05. *P < 0.05, **P < 0.01, ***P < 0.001, ****P < 0.0001. Exact *P*-values are provided in Supplementary Table S1.

*w*Ri has three *cif* gene pairs: two identical type 1 pairs and one type 2 pair (LePage *et al.* 2017; Lindsey *et al.* 2018). We first focus attention on the *cif_wRi[T1]_* genes, their protein sequence differences, and CI phenotype variation. Relative to CifA_*w*__Mel[T1]_, CifA_*w*__Ri[T1]_ protein has five amino acid substitutions in unannotated regions flanking the predicted domains (Figure 3A). One of these CifA substitutions is also present in CifA_*w*__Rec[T1]_. CifB_*w*__Ri[T1]_ has an in-frame stop codon introduced at residue 213 in the 1173-amino-acid-long protein (Figure 3A), and Glimmer 3 predicts that another protein coding sequence begins 16 amino acids later at residue 229. Thus, *cifB_wRi[T1]_* may yield two proteins: an N-terminal 212 amino acid protein and a C-terminal 945 amino acid protein. We refer to the gene sequences yielding the N-terminal and C-terminal peptides as *cifB_wRi[T1;N]_* and *cifB_wRi[T1;C]_*, respectively. Relative to CifB_*w*__Mel[T1]_, CifB_*w*__Ri[T1;N]_ has two amino acid substitutions, a seven amino acid N-terminal extension, and an early stop codon. In this region, CifB_*w*__Rec[T1]_ has the same sequence variations, excluding the early stop codon in addition to one extra substitution. CifB_*w*__Ri[T1;C]_ has three substitutions relative to CifB_*w*__Mel[T1]_: one in the first PDDEXK domain, a valine to methionine substitution marking the translation start site, and one in the unannotated region prior to the first PDDEXK domain (Figure 3A). In this C-terminal region, CifB_*w*__Rec[T1]_ shares all but the valine to methionine substitution. To investigate the genetic basis of wRi CI, we generated *cifA_wRi[T1]_*, *cifB_wRi[T1;N]_*, and *cifB_wRi[T1;C]_* transgenes. We additionally created a polycistronic *cifB_wRi[T1]_* transgene that expressed both the N-terminal and C-terminal peptides from a single transcript using a T2A sequence between the two proteins (Donnelly *et al.* 2001a,b). We refer to this polycistronic transgenic construct as *cifB_wRi[T1;poly]_*.

We first tested *cifA;B_wRi[T1;poly]_* males for their ability to induce CI and found that they did not reduce hatching (*P* = 0.55) (Figure 3B). Males dually expressing *cifA_wRi[T1]_* with either *cifB_wRi[T1;N]_* (*P* = 0.55; Supplementary Figure S3A) or *cifB_wRi[T1;C]_* (*P* = 0.32; Supplementary Figure S4A) also failed to reduce hatching, suggesting that dual expression of *cif_wRi[T1]_* transgenes cannot recapitulate CI. In addition, singly expressing *cifA_wRi[T1]_* (*P* > 0.99) or *cifB_wRi[T1;poly]_* (*P* > 0.99) males does not cause CI (Figure 3B). Next, to test if *cif_wRi[T1]_* genes can rescue strong *cif_wMel[T1]_* CI, we crossed *cifA;B_wMel[T1]_* males with *cifA_wRi[T1]_* (*P* > 0.99) and *cifA;B_wRi[T1;poly]_* (*P* > 0.99) females, both of which yielded hatching levels comparable to *cifA_wMel[T1]_* rescue (Figure 3B). These results indicate that *cifA_wRi[T1]_* is a rescue gene, and *cif_wRi[T1]_* transgenes do not cause CI when singly or dually expressed as cognate partners in *D. melanogaster*.

To further evaluate if *cif_wRi[T1]_* transgenes are capable of CI and whether variation in *cifA* or *cifB* may underpin the lack of CI above, we engineered and dually expressed non-cognate pairs of *cif_wRi[T1]_* genes with *cif_wMel[T1]_* genes. *cifA_wMel[T1]_*;*cifB_wRi[T1;poly]_* males did not yield a reduction in hatching compared to the compatible cross (*P* > 0.99; Figure 3C). Similarly, males dually expressing *cifA_wMel[T1]_* and either *cifB_wRi[T1;N]_* (*P* > 0.99; Supplementary Figure S3B) or *cifB_wRi[T1;C]_* (*P* > 0.99; Supplementary Figure S4B) did not reduce hatching. However, *cifA_wRi[T1];_cifB_wMel[T1]_* males caused near-complete embryonic death (Mdn = 0% hatching; *P* < 0.0001) that could be rescued by *cifA_wRi[T1]_* and *cifA_wMel[T1]_* females (Figure 3D). These findings suggest that *cifA_wRi[T1]_* contributes to both rescue and CI induction, but *cifB_wRi[T1]_* transgenes fail to contribute to CI.

### *Do the phylogenetic type 2* cif *genes from* w*Ri transgenically induce and rescue CI?*

**Figure 4:**
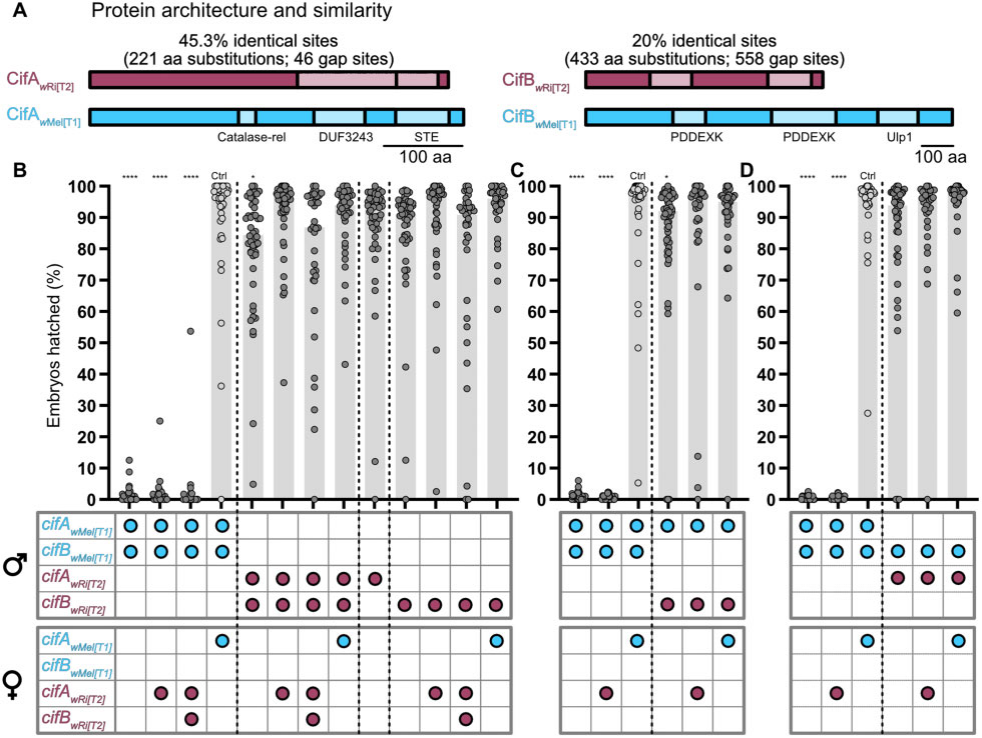
Cif_*w*Ri[T2]_ protein similarity and results of transgenic crosses including Cif_*w*Ri[T2]_ proteins. (A) Protein architecture of Cif_*w*__Mel[T1]_ and Cif_*w*__Ri[T2]_ (Lindsey *et al.* 2018). In an alignment of CifA_*w*__Mel[T1]_ and CifA_*w*__Ri[T2]_ (488 aa), there are 221 identical sites, 221 aa substitutions, and 46 gap sites. In an alignment of CifB_*w*__Mel[T1]_ and CifB_*w*__Ri[T2]_ (1239 aa), there are 248 identical sites, 433 aa substitutions, and 558 gap sites. Specific details on the kinds and locations of sequence variations are illustrated in Supplementary Figure S5. Hatch rate analyses testing (B) *cifA_wRi[T2]_*, *cifB_wRi[T2]_*, and *cifA;B_wRi[T2]_* for CI and rescue (*N* = 35–55 where each dot represents a clutch of embryos from a single mating pair), (C) *cifA_wMel[T1]_;cifB_wRi[T2]_* for CI (*N* = 39–56), and (D) *cifA_wRi[T2]_;cifB_wMel[T1]_* for CI (*N* = 31–45). Horizontal bars represent median embryonic hatching from single pair matings. Genotypes for each cross are illustrated below the bars where the genes expressed in each sex are represented by colored circles. Blue circles represent *cif_wMel[T1]_* genes and purple circles represent *cif_wRi[T2]_* genes. All flies were uninfected with *Wolbachia*. Each hatch rate contains the combined data of two replicate experiments, each containing all crosses shown. Asterisks above bars represent significant differences relative to a control transgenic rescue cross (denoted Ctrl) with an α = 0.05. *P < 0.05, **P < 0.01, ***P < 0.001, ****P < 0.0001. Exact *P*-values are provided in Supplementary Table S1.

Pairwise alignments of Cif_*w*__Mel[T1]_ and Cif_*w*__Ri[T2]_ proteins (488 and 1239 amino acids for CifA and CifB, respectively) reveal major divergence. First, CifA_*w*__Mel[T1]_ and CifA_*w*__Ri[T2]_ differ by 267 sites (45.3% identical sites), with 221 amino acid substitutions and 46 gap sites in the alignment (Figure 4A and Supplementary Figure S5). CifA_*w*__Ri[T2]_ has substitutions in all six of the sites that vary in CifA_*w*__Rec[T1]_ and CifA_*w*__Ri[T1]_, and two of the CifA_*w*__Ri[T2]_ substitutions are shared with both proteins, and a third is shared with CifA_*w*__Ri[T1]_. Second, CifB_*w*__Mel[T1]_ and CifB_*w*__Ri[T2]_ differ by 991 sites (20% identical sites), with 433 substitutions and 558 gap sites in the alignment (Figure 4A and Supplementary Figure S5). In addition, CifB_*w*Ri[T2]_ has substitutions in four of the six sites that vary in CifB_*w*Ri[T1]_ and CifB_*w*Rec[T1]_, but the specific amino acids are unique to CifB_*w*Ri[T2]_ (Supplementary Figure S5). Moreover, while the sequence lengths of the two CifA variants are comparable, CifB_*w*__Ri[T2]_ does not have the C-terminal Ulp1 domain that, for other distant type 1 Cif variants, acts *in vitro* as a deubiquitinase (Beckmann *et al.* 2017). It also has an eight-amino-acid N-terminal extension (Supplementary Figure S5), of which four amino acids are shared in the N-terminal extensions of CifB_*w*__Rec[T1]_ and CifB_*w*__Ri[T1]_.

First, we test if *cif_wRi[T2]_* transgenes cause and rescue CI in *D. melanogaster. cifA;B_wRi[T2]_* males caused a weak but statistically significant hatch rate reduction (Mdn = 84.4% hatching; *P* = 0.01; Figure 4B) that was rescued upon crossing with *cifA_wRi[T2]_* females (*P* > 0.99; Figure 4B). Similar to results with *cifA;B_wRec[T1]_* females above (Figure 2B), crossing *cifA;B_wRi[T2]_* males with *cifA;B_wRi[T2]_* females only slightly improved hatching such that it was no longer statistically different from the compatible control (Mdn = 86.9% hatching; *P* = 0.15); however, the median hatch rate was comparable when *cifA;B_wRi[T2]_* males were mated to uninfected females (Mdn = 84.4% hatching; Figure 4B). Thus, similar to *cif_wRec[T1]_*, it cannot be concluded that *cifA;B_wRi[T2]_* females are rescue-capable yet, but *cifA_wRi[T2]_* females clearly rescue *cifA;B_wRi[T2]_* CI as expected under the Two-by-One Model. In parallel, we showed that neither *cifA_wRi[T2]_* (*P* = 0.84) nor *cifB_wRi[T2]_* (*P* = 0.13) males alone reduce hatching, as expected (Figure 4B). These data suggest that *cifA;B_wRi[T2]_* males can cause very weak CI that can be rescued by *cifA_wRi[T2]_* females.

Next, we aimed to determine if the considerable intertype divergence between *cifA_wRi[T2]_* and *cifA_wMel[T1]_* underpins incompatibility between the strains (Figures 1C and 3A). Embryo death was observed when *cifA;B_wMel[T1]_* males mated with *cifA_wRi[T2]_* (Mdn = 0%; *P* < 0.0001) or *cifA;B_wRi[T2]_* (Mdn = 0%; *P* < 0.0001) females (Figure 4B), suggesting incompatibility between the gene variants. Reciprocally, embryonic hatching increased to compatible levels when *cifA;B_wRi[T2]_* males mated with *cifA_wMel[T1]_* females (*P* > 0.99) (Figure 4B). Together, these data suggest unidirectional CI between *cif_wMel[T1]_* and *cif_wRi[T2]_* such that *cifA_wMel[T1]_* can rescue CI caused by both variants, but *cifA_wRi[T2]_* can only rescue its own lethality. This is the first empirical finding that type 1 and 2 *cif* genes are partially compatible and thus likely share similar CI mechanisms.

Finally, as previously done for *cif_wRec[T1]_* and *cif_wRi[T1]_*, we combinatorially tested if non-cognate expression of *cif_wRi[T2]_* and *cif_wMel[T1]_* genes underpins the genetic basis of CI level variation. First, *cifA_wMel[T1]_*;*cifB_wRi[T2]_* males yielded a small but significant hatch rate reduction (Mdn = 92.0% hatching; *P* = 0.01), relative to the compatible control. Second, *cifA_wRi[T2]_* (*P* > 0.99) and *cifA_wMel[T1]_* (*P* = 0.40) females rescued the weak hatch rate reduction (Figure 4C). Finally, *cifA_wRi[T2]_*;*cifB_wMel[T1]_* males had a similar, but statistically insignificant, impact on hatching (*P* = 0.07) relative to *cifA_wMel[T1]_*;*cifB_wRi[T2]_* males (Figure 4D). Thus, dual expression of both non-cognate pairs yields a small reduction in hatching, and weak *cifA_wMel[T1]_*;*cifB_wRi[T2]_* CI was rescuable. Contrary to non-cognate expression of *cifA_wRec[T1]_* or *cifA_wRi[T1]_* with *cifB_wMel[T1]_*, neither non-cognate pairing of *cif_wRi[T2]_* and *cif_wMel[T1]_* yielded strong CI. These data again suggest that divergent *cif* types can work together to cause a weak CI-like phenotype.

## Discussion

The Two-by-One genetic model of CI states that *cifA;B* males cause CI, and *cifA* females rescue that CI (Shropshire and Bordenstein 2019). However, it remains unknown if this model can be widely generalized across *cif* variants. Likewise, it is unknown whether *cif* variation alone explains incompatibilities between *Wolbachia* strains and CI level variation. Here, we use transgenic tools in *D. melanogaster* to test if *cif* homologs from *w*Rec and *w*Ri contribute to CI and rescue, whether *cif* genetic variation relates to strain incompatibility (Charlat *et al.* 2001; LePage *et al.* 2017; Shropshire *et al.* 2018; Bonneau *et al.* 2018, 2019), and if *cif* sequence variation determines transgenic CI levels.

**Figure 5:**
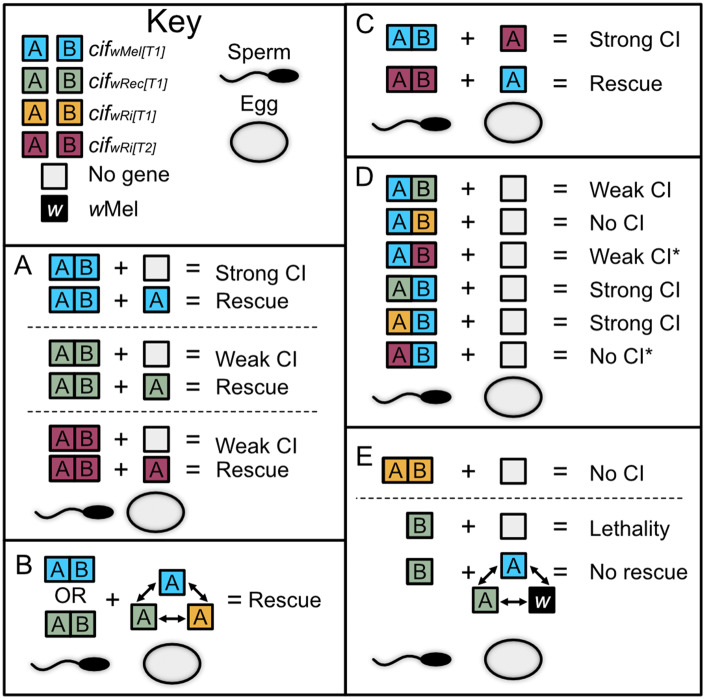
Summary of findings. (A) *cif_wRec[T1]_* and *cif_wRi[T2]_* induce CI phenotypes in a manner consistent with the Two-by-One genetic model of CI previously established with *cif_wMel[T1]_* genes (Shropshire and Bordenstein 2019). (B) CI induced by type 1 *cif* pairs can be interchangeably rescued by *cifA_wMel[T1]_*, *cifA_wRec[T1]_*, and *cifA_wRi[T1]_* transgene expressing females. (C) Unidirectional CI is caused between *cif_wRi[T2]_* and *cif_wMel[T1]_* transgenes such that *cifA_wMel[T1]_* can rescue type 2 transgenic CI but *cifA_wRi[T2]_* fails to rescue type 1 transgenic CI. (D) Dual non-cognate expression of type 1 homologs reveals that *cifB* homologs cause weak or no CI while *cifA* homologs can contribute to strong transgenic CI. Non-cognate pairs that cause CI can be rescued by *cifA*-expressing females. Dual non-cognate expression of type 1 and 2 *cif* homologs reveals that despite amino acid and domain divergence, they may functionally work together to induce weak or marginal CI. * denotes significant or nearly significant levels of very weak CI. (E) *cif_wRi[T1]_* do not contribute to CI and *cifB_wRec_* causes complete embryonic death that cannot be rescued by *cifA_wRec_*, *cifA_wMel_*, or *w*Mel-infected females.

We report four key findings (Figure 5): (i) Evidence is consistent with a Two-by-One genetic basis for rescuable CI, but only weak CI is caused by *cif_wRec[T1]_* and *cif_wRi[T2]_* homologs (Figure 5A). (ii) Both type 1 *cifA* homologs rescue strong *cifA:B_wMel[T1]_* CI (Figure 5B), supporting the hypothesis that closely related *cif* genes are compatible (Charlat *et al.* 2001; LePage *et al.* 2017; Shropshire *et al.* 2018; Bonneau *et al.* 2018). (iii) Type 2 *cifA_wRi[T2]_* homologs cannot rescue *cifA;B_wMel[T1]_* CI, but the type 1 *cifA_wMel[T1]_* can rescue weak *cifA;B_wRi[T2]_* CI (Figure 5C), suggesting that different *cif* types may mechanistically work together, and genetic distance may contribute to unidirectional CI instead of the simple expectation of bidirectional CI. (iv) Type 1 *cifB* genetic variants determine CI level variation when paired with *cifA_wMel[T1]_* whereas both type 1 *cifA* homologs contribute to strong CI when paired with *cifB_wMel[T1]_* (Figure 5D). We also report two results contrary to our initial predictions: *cifB_wRec[T1]_* males yield unrescuable sperm infertility or embryonic death, and *cifB_wRi[T1]_* does not induce transgenic CI alone or with any *cifA* variant (Figure 5E). Below we interpret these findings in the context of the *cif* genotype–phenotype relationship for CI level variation, incompatibility relationships between *Wolbachia* strains, *cif* genotype by host genotype interactions, and CI mechanisms.

### *The genetic basis of* w*Rec (type 1) and* w*Ri (type 2) CI and rescue*

*w*Rec and *w*Ri induce strong CI in their native hosts (Turelli and Hoffmann 1991; Werren and Jaenike 1995; Shoemaker *et al.* 1999), leading to the prediction that their corresponding *cif* genes could yield strong transgenic CI in *D. melanogaster*. However, a small but significant and repeatable CI was observed when *cifA;B_wRec[T1]_* and *cifA;B_wRi[T2]_* were expressed in uninfected *D. melanogaster* males, and that CI was rescued by females expressing their cognate *cifA* or *cifA_wMel[T1]_*. Thus, we conclude that these gene pairs function in accordance with the Two-by-One genetic model of CI (Shropshire and Bordenstein 2019). Moreover, this is the first report of a CI-like phenotype caused by the phylogenetic type 2 *cif* genes. However, it is important to emphasize that a firm conclusion about the full genetic determinants of CI and rescue for these gene pairs is inhibited by the weakened CI levels. Unlike *cifA;B_wRec[T1]_* and *cifA;B_wRi[T2]_*, dual expression of *cifA_;_B_wRi[T1]_* failed to cause CI. We propose three non-exclusive hypotheses for why weak CI is induced by *cif_wRec[T1]_* and *cif_wRi[T2]_* transgenes, and we discuss interpretations for why *cifA;B_wRi[T1]_* males fail to cause CI, and why *cifB_wRec[T1]_* alone causes embryonic death.

First, strong transgenic CI can be impacted by the method of transgenic expression. Indeed, the first report of transgenic *w*Mel CI with *cifA;B_wMel[T1]_* expression in males revealed incomplete CI (LePage *et al.* 2017), and later optimization of the expression driver was necessary to cause consistently strong transgenic CI (Shropshire and Bordenstein 2019). Here, we used the expression system optimized for transgenic expression of *w*Mel *cif* genes (Shropshire and Bordenstein 2019), and thus, it is plausible that the level or location of expression optimal for *w*Mel-induced CI is not the same as for these other gene products. Second, other genes may be necessary to cause strong CI alongside *cif_wRec[T1]_* and *cif_wRi[T2]_* genes. These may include additional *cif* gene copies or other *Wolbachia* and prophage WO genes. For instance, *w*Ri contains both type 1 and type 2 *cif* genes (LePage *et al.* 2017), and all *Wolbachia* strains known to carry type 2 *cifs* also harbor genes from other *cif* types (LePage *et al.* 2017; Lindsey *et al.* 2018). Thus, co-expression of both *cif* types may be necessary to cause strong CI, or additional genes predicted to interact with eukaryotes may modulate CI (Wu *et al.* 2004; Yamada *et al.* 2011; Bordenstein and Bordenstein 2016). Third, several transinfection and introgression studies show that host genotype affects CI levels (Poinsot *et al.* 1998; Bordenstein *et al.* 2003). The proximal basis of this affect remains unknown, but it is predicted to be related to *Wolbachia* titers and *cif* expression profiles (Shropshire *et al.* 2020b). For instance, *w*Mel is considered as a weak CI inducer, but strict control of several variables that covary with *Wolbachia* titers and *cif* expression enables strong CI (Reynolds and Hoffmann, 2002, Yamada *et al.* 2011, Layton *et al.* 2019). Moreover, strong *w*Mel transgenic CI is possible (Shropshire *et al.* 2019), thus suggesting that a weak CI strain can cause strong transgenic CI. However, while titer and *cif* gene expression likely control CI strength within a system, it is plausible that Cif amino acid sequence also corresponds with a change in efficiency when binding to *D. melanogaster* targets in a heterologous expression system.

In summary, while these data are currently in line with the Two-by-One genetic model of CI, optimization of the transgenic expression system in *D. melanogaster* (Duffy 2002) will be necessary to confirm that these genes can recapitulate strong CI and rescue. If optimization fails to improve the pentrance, then other proteins may modulate the phenotypic potency of CI and be required for strong CI. Alternatively, homologous proteins may not efficiently bind to targets in other hosts, preventing strong CI under heterologous expression. Notably, since non-cognate expression of *cifA* homologs with *cifB_wMel[T1]_* yielded strong CI, it is clear that *cifA* sequence variation is not responsible for weakened CI. This is perhaps unsurprising given that mutagenesis assays of Cif_*w*Mel[T1]_ proteins reveal that CI expression is more likely to be impacted by mutations in CifB than in CifA (Shropshire *et al.* 2020a). Thus, the aforementioned effects of suboptimal expression, need for additional genes, or inefficient binding to *D. melanogaster* targets could be related to the expression of *cifB* homologs.

Similarly, *cifA;B_wRi[T1]_* males do not cause CI, but notably non-cognate dual expression with *cif_wMel[T1]_* genes revealed that *cifA_wRi[T1]_*, but not *cifB_wRi[T1]_*, contributes to strong CI. This result is perhaps expected since *cifB_wRi[T1]_* has an early in-frame stop codon relative to *cifB_wMel[T1]_* that contributes to its annotation in the *w*Ri genome as a nonfunctional pseudogene. Despite this, we hypothesized that *cifB_wRi[T1]_* would contribute to CI since *w*Ri’s expression of both type 1 and 2 *cif* genes aligns with the patterns of unidirectional CI between *w*Ri and *w*Mel (Figure 1D). We provide four hypotheses to explain the absence of CI under *cifA;B_wRi[T1]_* expression. First, *cifB_wRi[T1]_* is a pseudogene and is not capable of contributing to CI. Second, since *w*Ri harbors two identical type 1 gene pairs and a type 2 gene pair (LePage *et al.* 2017), both type pairs may be required for complete CI expression. Third, the early stop codon in *cifB_wRi[T1]_* may not prevent translation of the full-length protein since some stop codons slow translation instead of halting it (Wangen and Green 2020). Thus, a full-length CifB_*w*__Ri[T1]_ protein may be generated despite the internal stop codon, and we did not test that here. Finally, to co-express the N-terminal and C-terminal CifB_*w*__Ri[T1]_ proteins, we introduced a sequence between the two proteins that causes translational slippage and multi-protein translation from a single transcript (Donnelly *et al.* 2001a,b). This method yields a C-terminal sequence extension to the first protein that may alter protein function. In summary, these data currently support pseudogenization of the *cifB_wRi[T1]_* gene, but transgenic optimization and co-expression with other *cif* proteins will be necessary to fully rule out alternative explanations.

Contrary to initial predictions, *cifB_wRec[T1]_* transgenic males cause sperm infertility and/or embryonic death when mated with uninfected females. At its surface, *cifB_wRec[T1]_* alone may be interpreted to cause CI. However, this lethality is not rescued by *cifA_wRec[T1]_*, *cifB_wMel[T1]_*, or *w*Mel females, and it associates with unusual cytological defects relative to wMel transgenic CI. As bona fide CI is defined by male embryonic lethality, a standard set of cytological defects, and the ability to rescue them, we do not interpret *cifB_wRec[T1]_* lethality as CI. However, it is plausible that a *w*Rec-infected fly may rescue *cifB_wRec[T1]_* lethality. Since testing this requires difficult transinfection of *w*Rec into *D. melanogaster*, we did not test this hypothesis. Conversely, *cifA:B_wRec[T1]_* males also weakly reduce hatching that is rescuable by *cifA_wRec[T1]_* and *cifA_wMel[T1]._* Thus, these data suggest that while *cifB_wRec[T1]_* alone may cause an unusual lethality, a CI-like phenotype is only achieved when CifA and CifB proteins are dually expressed in males. We discuss our mechanistic interpretations of the results below (see “CI mechanism” section).

### Incompatibility relationships

*w*Mel and *w*Ri *Wolbachia* are unidirectionally incompatible when *w*Mel *Wolbachia* are transinfected into *D. simulans* (Poinsot *et al.* 1998) (Figure 1C). Specifically, *w*Ri rescues *w*Mel CI, but *w*Mel does not rescue *w*Ri CI. We hypothesized that cif sequence and copy number variation controls these incompatibility relationships (LePage *et al.* 2017). Since *w*Ri has both type 1 and 2 *cif* genes, we expected *cifA_wRi[T1]_* to rescue *cifA;B_wMel[T1]_*-induced CI because the *cifA* variants are closely related, and *cifA_wMel[T1]_* would not rescue *cifA;B_wRi[T2]_*-induced CI because *cifA_wMel[T1]_* is highly divergent from the type 2 gene pair (LePage *et al.* 2017) (Figure 1D). In addition, *w*Rec and *w*Mel have only type 1 genes with a few amino acid changes, leading to the prediction that they are compatible (Figure 1C and D). We tested three key predictions of this *cif* genotype—CI phenotype hypothesis: (i) *cifA_wRi[T1]_* rescues transgenic *cif_wMel[T1]_* CI , (ii) *cifA_wRi[T1]_* cannot rescue transgenic *cif_wMel[T1]_* CI, and (iii) *cifA_wMel[T1]_* cannot rescue transgenic *cif_wRi[T2]_* CI.

As predicted, type 1 *cifA_wRec[T1]_* and *cifA_wRi[T1]_* can each rescue transgenic *cifA;B_wMel[T1]_* CI. In addition, *cifA_wRi[T2]_* cannot rescue *cifA;B_wMel[T1]_* CI, despite being able to rescue *cifA;B_wRi[T2]_* CI. These data align with expectations that only closely related *cif* homologs are compatible (Figure 1D). However, we also hypothesized that *cifA_wMel[T1]_* does not rescue *cifA;B_wRi[T2]_* CI, but rescue occurred at the same levels for both *cifA_wMel[T1]_* or *cifA_wRi[T2]_* females, suggesting that both *cifA* variants were capable of rescuing transgenic *cifA;B_wRi[T2]_* CI. These results imply a unidirectional incompatibility between type 1 and type 2 genes where type 1 genes cannot be rescued by type 2 genes, but the reciprocal cross is compatible. Not only are these results contrary to our expectations, but they also fail to sufficiently explain the reported unidirectional CI between *w*Mel and *w*Ri since rescue occurs in the opposite direction than we report here (Poinsot *et al.* 1998). We propose two possible explanations for these results.

First, host genotype may impact incompatibility relationships. Two studies evaluated the CI relationships between *w*Mel and *w*Ri, revealing unidirectional CI when wMel is transinfected into *D. simulans* (Poinsot *et al.* 1998) and no incompatibility when *w*Mel-infected *D. melanogaster* is crossed with *w*Ri-infected *D. simulans* (Gazla and Carracedo 2009). Similarly, two *Wolbachia* from the *Nasonia longicornis* parasitoid wasp switched from being unidirectionally to bidirectionally incompatible when moved into the same genetic background (Raychoudhury and Werren 2012). Thus, there is support for host control of *Wolbachia* reproductive parasitism and incompatibility relationships. It is unknown what kind of incompatibility relationships might occur if both *w*Mel and *w*Ri are in a *D. melanogaster* host background. However, our transgenic *cif* expression data suggest that *w*Mel can rescue *w*Ri, but not vice versa. Thus, we hypothesize that rescue, in particular, is impacted by host genotype such that *cifA* expressed natively (*e.g. w*Mel in *D. melanogaster* or *w*Ri in *D. simulans*) has expanded rescue capability as compared to expression in introduced strains. This hypothesis can be tested through transinfection of *w*Ri into a *D. melanogaster* background or through transgenic expression of *cif_wMel[T1]_*, *cif_wRi[T1]_*, and *cif_wRi[T2]_* in *D. simulans*. Second, it remains possible that there are dynamic interactions between Cifs such that multiple phylogenetic types interact with one another to impact the phenotypic output. For instance, since *w*Ri naturally maintains both type 1 and 2 *cif* genes (LePage *et al.* 2017; Lindsey *et al.* 2018), expression of both may be required to induce the reported compatibility relationships between *w*Mel and *w*Ri (Poinsot *et al.* 1998). This hypothesis can be tested through the dual expression of both types 1 and 2 gene pairs and crossing to *cif_wMel_* expressing flies.

### CI mechanism

The cellular and molecular bases of CifA and CifB in CI remain an active area of investigation. To date, *in vitro* assays determined that CifB_*w*__Mel[T1]_ and CifB_*w*__Pip[T1]_ act in part as deubiquitinases, CifB_*w*__Pip[T4]_ acts in part as a nuclease, cognate CifA;B pairs of *w*Mel and *w*Pip can bind, and both CifA and CifB interact with host proteins when transgenically expressed in *D. melanogaster* (Beckmann *et al.* 2017, 2019c; Chen *et al.* 2019). There are two mechanistic models for CI that are currently debated: host modification (HM) and toxin antidote (TA) (Hurst 1991; Poinsot *et al.* 2003; Shropshire *et al.* 2019; Beckmann *et al.* 2019a,b). HM models posit that CifA;B proteins cause CI by modifying host factors during spermatogenesis, and those modifications are transferred to the embryo. Rescue occurs when CifA in females reverses those sperm modifications in the embryo (Shropshire *et al.* 2018, 2019). Conversely, TA models suggest that CifB is transferred to the embryo via the sperm and kills the embryo unless its lethality is rescued through binding to CifA in the embryo (Beckmann *et al.* 2019a; Shropshire *et al.* 2019). Notably, there is no evidence of paternal transfer of Cif toxin(s), and it remains unclear whether CifA-B binding is related to CI or rescue (Shropshire *et al.* 2019). Thus, current data are insufficient to support one model over the other. Here, we place three findings above into the context of CI’s mechanistic basis: (i) CifB sequence variation impacts CI level variation, (ii) closely related type 1 CifA can be interchanged for both CI and rescue, and (iii) CifB_*w*__Rec[T1]_ induces complete embryonic death when singly expressed.

A key finding of this study is that *cifB_wRec[T1]_* and *cifB_wRi[T1]_* sequence variation impacts *cifA;B*-induced CI level when transgenically expressed in *D. melanogaster*. We propose two mechanistic explanations. First, foreign CifB homologs in a new host may be less efficient or unable to bind host proteins or to CifA. Proteomic analyses of synthesized Cif_*w*__Pip[T1]_ proteins bound to a column and washed with *D. melanogaster* lysate revealed that CifB_*w*__Pip[T1]_, CifA_*w*__Pip[T1]_, or CifA;B_*w*__Pip[T1]_ proteins bind between 15 and 60 fly proteins (Beckmann *et al.* 2019c). The sheer number of potential CifB-binding partners may contribute to the large impact of *cifB_wRec[T1]_* and *cifB_wRi[T1]_* sequence variation on CI levels. Alternatively, *cifB_wRec[T1]_* and *cifB_wRi[T1]_* sequence variation may contribute to variation in its tissue localization, subcellular localization, or ability to diffuse between cellular components. CI levels have been correlated with the number of *Wolbachia*-infected spermatocytes and spermatids during spermatogenesis in wRi-infected *D. simulans* (Clark and Karr 2002; Veneti *et al.* 2003; Clark *et al.* 2003), but even uninfected spermatocytes often result in modified sperm that can cause CI (Riparbelli *et al.* 2007), suggesting that CifA and/or CifB are diffusible between spermatocytes or during earlier stages of spermatogenesis. Binding and localization studies would elucidate these hypotheses.

While *cifB_wRec[T1]_* and *cifB_wRi[T1]_* sequence variation clearly impacts the CI level in transgenic *D. melanogaster*, type 1 *cifA_wReci[T1]_* and *cifA_wRi[T1]_* homologs were notably interchangeable and contribute to both strong CI and rescue. These data importantly suggest that while *cifB_wRec[T1]_* and *cifB_wRi[T1]_* sequence variation may be specifically attuned to a distinct host background, transgenic CifA is less subject to variation in host background. For instance, it is plausible that while CifB is interacting with rapidly evolving host targets in an arms race, CifA interacts with a set of conserved targets. One prediction of this hypothesis would be that CifA would be under purifying selection to retain compatibility with conserved host targets. Indeed, comparative sequence analyses reveal not only that type 1 CifAs are under strong purifying selection (Shropshire *et al.* 2018), but also that CifA sequence length is highly conserved across the phylogenetic types (LePage *et al.* 2017; Lindsey *et al.* 2018) and less prone to pseudogenization than CifB (Martinez *et al.* 2020). Thus, a type of HM model could be proposed whereby CifB acts simply as an “accessory” to bind CifA and unlock its access to conserved host processes not otherwise accessible in the absence of CifB. In addition, theory suggests that hosts will evolve resistance to CI while maintaining rescue (Turelli 1994), and many of the same predictions above would also apply in this scenario. For instance, if CifA’s targets in rescue and CI are similar, then one would predict the conservation of those targets to maintain rescue, while also maintaining CifA’s ability to contribute to CI. However, variation in CifB’s targets would only inhibit CI induction; thus, selection may favor variation in CifB targets to develop resistance against CI.

Finally, *cifB_wRec[T1]_* males cause complete infertility and/or embryonic death, but these defects are not rescuable and associate with unusual cytological defects. As such, *cifB_wRec[T1]_*-induced effects are not consistent with our expectations for CI induction. We propose two hypotheses for these results. First, CifB may cause CI in the absence of CifA. Singly expressing *cifB* homologs in yeast causes temperature-sensitive lethality that can be reduced when dually expressed with cognate *cifA* (Beckmann *et al.* 2017, 2019a,b; Chen *et al.* 2019). However, aside from singly expressing *cifB_wRec[T1]_* in this study, only *cifB_wPip[T4]_* males cause weak embryonic lethality (Chen *et al.* 2019), but there is also no evidence that *cifB_wPip[T4]_*-induced lethality can be rescued; moreover, more embryonic death is induced when *cifB_wPip[T4]_* is dually expressed with *cifA_wPip[T4]_* (Chen *et al.* 2019). Thus, *cifB_wRec[T1]_*-induced lethality varies from these historical results because *cifB_wRec[T1]_* yields near-complete embryonic death that is weakened and becomes rescuable only when dually expressed with *cifA_wRec[T1]_*. It is plausible that the reduced embryonic death from *cifA;B_wRec[T1]_* relative to *cifB_wRec[T1]_* alone is explained by *cifA* protection of a *cifB*-mediated sperm toxicity. However, it then becomes unclear why embryonic death increases in all other cases of dual transgene expression in insects and why *cifB_wRec[T1]_* is the only *cifB* homolog to cause near-complete embryonic death. Second, *cifB_wRec[T1]_* embryonic lethality may be a transgenic, off-target artifact. CifA’s binding to CifB (Beckmann *et al.* 2017) may be required for proper function, such as localizing CifB to its cellular target or priming its activity (Shropshire *et al.* 2019). Thus, in the absence of CifA_*w*__Rec[T1]_, CifB_*w*__Rec[T1]_ may result in off-target enzymatic activity and/or disruption of crucial host processes unrelated to CI induction, thus leading to a sterility independent of CI. This may explain why CifB_*w*__Rec[T1]_ defects cannot be rescued. However, why would CifB_*w*__Rec[T1]_ cause artifactual embryonic death when singly expressing other CifB homologs does not? CifB_*w*__Rec[T1]_ has a unique C-terminal truncation beyond the putative deubiquinase domain. Numerous insecticidal toxins and bacterial protoxins have C-terminal self-inhibitors that prevent enzymatic activity, including latrotoxins (Rohou *et al.* 2007), Cry toxins (Peña-Cardeña *et al.* 2018), and botulinum neurotoxins (Mizanur *et al.* 2013). As such, some CifB proteins may contain C-terminal self-inhibitors that prevent their action in males. If CifB_*w*__Rec[T1]_ lacks this self-inhibitor, then its activity would not require cleavage. When expressed by *Wolbachia*, this toxicity may not be observed if the expression profile is tightly regulated or if other proteins are expressed that suppress CifB_*w*__Rec[T1]_ function. Support of these hypotheses will require the characterization of CifB’s C-terminus and the functional role of CifA-B binding. In summary, *cifB_wRec[T1]_*-induced effects are of interest, but significant caution is warranted as this lethality is not rescuable, which is a requirement for bona fide CI.

### Summary

Here, we set out to investigate the hypothesis that *cif* sequence variation directly relates to CI phenotypic variation by evaluating cognate combinations of the *cif* genes and their incompatibility relationships. Moreover, we engineered non-cognate gene sets to test CI capacity and links between *cif* sequence variation and variation in CI level. In summary, we determined for the first time that type 1 *cif* homologs from *w*Rec and type 2 *cif* homologs from *w*Ri cause weak CI when transgenically expressed in *D. melanogaster*, variation in *cifB* contributes to CI level variability, divergent *cifA* fails to rescue transgenic *cifA;B_wMel[T1]_* CI, and type 1 *cifA* homologs are interchangeable for inducing both strong CI and rescue. We discuss these results in the context of CI’s Two-by-One genetic basis in *w*Rec and *w*Ri, incompatibility relationships, and CI mechanism. The work expands upon our understanding of the genetics of CI and incompatibilities between *Wolbachia* strains, and they establish novel hypotheses regarding the *cif* mechanism, CI level variation, and the relationship between CI phenotypes and host genetics.
